# The Association between Parental Psychological Control, Deviant Peer Affiliation, and Internet Gaming Disorder among Chinese Adolescents: A Two-Year Longitudinal Study

**DOI:** 10.3390/ijerph17218197

**Published:** 2020-11-06

**Authors:** Shuang Lin, Chengfu Yu, Jun Chen, Jing Sheng, Yousong Hu, Lin Zhong

**Affiliations:** 1 School of Psychology, South China Normal University, Guangzhou 510631, China; 2019010200@m.scnu.edu.cn (S.L.); 2020010232@m.scnu.edu.cn (J.S.); 2018010208@m.scnu.edu.cn (Y.H.); 2019022997@m.scnu.edu.cn (L.Z.); 2 Key Laboratory of Brain, Cognition and Education Sciences (South China Normal University), Ministry of Education, South China Normal University, Guangzhou 510631, China; 3 Center for Studies of Psychological Application, South China Normal University, Guangzhou 510631, China; 4 Guangdong Key Laboratory of Mental Health and Cognitive Science, South China Normal University, Guangzhou 510631, China; 5 Department of Psychology and Research Center of Adolescent Psychology and Behavior, School of Education, Guangzhou University, Guangzhou 510006, China; yuchengfu@gzhu.edu.cn

**Keywords:** Internet gaming disorder, parental psychological control, deviant peer affiliation, longitudinal research, adolescent

## Abstract

Abundant empirical research indicates a relationship between parental psychological control and adolescent Internet gaming disorder (IGD), but the direction and underlying mechanism of this association remain unclear. Using a two-year longitudinal design across three time points, the present study examined the reciprocal processes between parental psychological control and IGD and explored whether deviant peer affiliation explains this bidirectional association. The sample consisted of 908 participants (480 boys and 428 girls) who participated in three measurements and completed questionnaires assessing parental psychological control, deviant peer affiliation, and IGD. Autoregressive cross-lagged models indicated a direct reciprocal relationship between parental psychological control and IGD. Furthermore, the results showed that parental psychological control exerts an indirect effect on adolescent IGD via deviant peer affiliation, but the inverse indirect effect via deviant peer affiliation was non-significant. Knowledge regarding the direct and underlying mechanisms of the reciprocal relationship between parental psychological control and IGD has important implications for prevention and intervention of adolescent IGD.

## 1. Introduction

With the passing of time and technological progress, individual lifestyles and behavior patterns are constantly evolving. Individual leisure activities and communication have changed, which may lead to new psychological health problems. Internet gaming disorder (IGD) is a subtype of Internet addiction and is defined as the “uncontrollable, excessive, and compulsive use of online games that causes serious problems such as social and/or emotional problems” [1]. Due to different socio-cultural backgrounds, different diagnostic criteria, and different regions, the prevalence rates of IGD vary [2]. The prevalence of IGD ranged from 0.7% to 15.6% in international samples without a defined age range [3] and was as high as 3.5% to 17% in the Chinese sample [4]. As IGD has become a global public health issue, it is now incorporated in the updated version of the DSM-5 and is thus attracting great attention from researchers [5]. Adolescents are vulnerable to various types of addictive behaviors because their brains have not fully matured [6] and they face multiple pressures in social, psychological, and academic areas [7]. In addition, the adverse effects of addictive behavior have been demonstrated to continue throughout adulthood [8]. IGD has been widely reported to affect adolescents’ cognitive functioning as well as their mental and physical health, contributing to negative outcomes, such as academic failure, lack of self-confidence, social anxiety, and poor sleep quality [9,10,11,12]. To develop effective prevention and intervention programs for treating IGD, it is essential to explore the risk factors and clarify the mediating mechanisms of adolescent IGD.

An increasing number of empirical studies indicate that parental psychological control is closely related to adolescent Internet addiction, which is not limited to IGD [11,13,14]. However, previous research has focused on the unidirectional influence of parental psychological control on adolescent IGD, and little is known about the bidirectional nature of this association and its underlying mechanism. Only a bidirectional model can fully describe the complex connections between parents and adolescents [15,16]. Moreover, traditional Chinese culture emphasizes the absolute obedience of children to their elders [17], which leads to more frequent use of psychologically controlling practices by parents in Chinese culture [11,18]. In this context, we constructed an autoregressive cross-lagged model to examine the direct and reciprocal association between parental psychological control and adolescent IGD as well as the potential mediating mechanisms among Chinese adolescents to better understand these developmental processes and provide valuable information to inform the design of effective prevention and intervention programs.

### 1.1. Parental Psychological Control and Adolescent IGD

Parental psychological control refers to the use of pressure to control children’s behavior by overly regulating their thoughts and/or feelings, which interferes with their ability to develop identity and autonomy [19]. Self-determination theory (SDT) [20] suggests that self-determination either promotes or weakens an individual’s intrinsic motivation and internalization, which could explain the developmental association between parental psychological control and IGD. Przybylski et al. demonstrated that Internet games rapidly fulfill adolescents’ basic psychological needs for autonomy, competence, and relatedness [21]. Hence, we can speculate that parental psychological control may facilitate IGD from the perspective of the SDT framework. Adolescence is a developmental period characterized by the search for autonomy and competence, and parental psychological control intrudes upon children’s basic psychological needs [13,22], which could result in a tendency toward addiction to online games that satisfy those needs [23]. Therefore, the violation of adolescents’ basic psychological needs through parents’ use of psychological control might contribute to adverse developmental outcomes, such as IGD. Indeed, a large body of cross-sectional empirical research indicates that parental psychological control is positively associated with adolescent Internet addiction, including IGD [11,13,24]. Koo and Kwon posited that adolescents who experience Internet addiction and IGD display similar psychological symptoms, which are different manifestations of the same underlying vulnerability [25]. Furthermore, a longitudinal study involving 3328 students in Hong Kong revealed that parental psychological control positively predicted subsequent Internet addiction among adolescents [14].

Furthermore, Patterson’s social coercion theory [26,27], which emphasizes that parental rearing patterns and adolescent behaviors interact and affect each other, may explain the reciprocal association between parenting and adolescent behaviors. Specifically, not only do parental rearing patterns affect adolescent behaviors, but adolescent problem behaviors can also lead to changes in parenting behaviors [15,16,26]. Research has documented a bidirectional association between parenting behaviors (parental behavior control and Internet-specific parenting) and adolescent IGD [28,29]. For example, a longitudinal study using two-wave data indicated that adolescents who exhibit IGD could elicit ineffective parental responses, which, in turn, further exacerbate adolescent IGD [29]. More importantly, Su et al., who conducted a three-wave longitudinal study using a sample of 1490 Chinese adolescents, demonstrated the reciprocal association between parental behavior control and adolescent IGD [28]. However, to the best of our knowledge, thus far, no study has examined the reciprocal relationship between parental psychological control and the development of IGD among adolescents. 

It is also worth noting that Chinese culture is influenced by traditional Confucian philosophy which emphasizes children’s full respect toward and obedience to their parents [16,17]. Deeply influenced by Confucian culture, Chinese parents may tend to adopt controlling parenting styles, such as psychological control [11,18]. Moreover, there is a Chinese proverb, namely “learning is above doing any other things”. Chinese parents devote considerable attention to the education of their children; in China, under these cultural values, possessing knowledge is deemed as the ability to change one’s fate and determine one’s future academic achievements. Thus, Chinese parents are very concerned about the academic performance of their children [30,31] and things that are irrelevant to learning may not be allowed. Therefore, we speculate that when adolescents spend significant time engaged in online games, their learning time may be reduced, which, in turn, will elicit more frequent use of parental psychological control. Based on these findings, we aimed to test the bidirectional association between parental psychological control and IGD among Chinese adolescents.

### 1.2. Mediating Role of Deviant Peer Affiliation

Adolescence is an important period of separation, which is characterized by the search for autonomy and independence. Parental psychological control interferes with the basic psychological needs of adolescents [22], which may lead them to seek intimacy and support from peers [32] and may increase the likelihood of their affiliation with deviant adolescents [33]. A cross-sectional study in China revealed that parental psychological control is associated with increased risk of deviant peer affiliation [34]. Additionally, a longitudinal study involving 497 Dutch adolescents demonstrated that excessive parental supervision does not fulfill adolescents’ basic psychological needs and positively predicts affiliation with deviant peers [35]. Therefore, it is likely that parental psychological control positively predicts subsequent deviant peer affiliation.

Previous research has highlighted that deviant peer affiliation is an important predictive factor for shaping adolescent IGD [23,34]. Adolescents are influenced by deviant peers through observational learning and behavior modeling as well as peer pressure [36], which increases the likelihood of disapproved behaviors, which may result in social rejection. Moreover, one study suggests that the appeal of Internet gaming could rapidly satisfy individuals’ psychosocial needs, as adolescents can achieve virtual success in online games to escape the interpersonal stress of real life [37]. Furthermore, some longitudinal studies reveal that deviant peer affiliation is a mediator between family factors and adolescent externalizing problems [23,38]. For example, a one-year longitudinal study demonstrated that poor parent–adolescent relationships could promote adolescent IGD by means of increasing deviant peer affiliation [23]. Thus, we speculate that deviant peer affiliation acts as a mediator in the path from parental psychological control to adolescent IGD. 

Deviant peer affiliation may act as a mediator of the bidirectional longitudinal association between parental psychological control and adolescent IGD. Specifically, not only does deviant peer affiliation play a mediating role in the path from parental psychological control to adolescent IGD but also in the path from IGD to parental psychological control. Adolescents select into peer groups via homophily selection and default selection [39], which could explain the association between IGD and deviant peer affiliation. Adolescents with IGD actively seek affiliation with peers who exhibit similar behavior via homophily selection [40]. Moreover, because of isolation from or rejection by mainstream peers [41], adolescents affiliate with deviant peers due to default selection, which occurs passively because of a lack of viable alternatives [42]. Some longitudinal empirical research has demonstrated the reciprocal link between externalizing problems and deviant peer affiliation [31,43]. Additionally, from the perspective of Patterson’s social coercion theory [26,27], when adolescents exhibit deviant behavior (i.e., deviant peer affiliation), parents may become stricter with their children and attempt to force them to change their behavior. Conversely, Dishion et al. indicate that as adolescents increasingly affiliate with deviant peers, their parents may give up on disciplining their children [40]. These two viewpoints require further elucidation. Nevertheless, there is currently no research that has tested whether deviant peer affiliation predicts subsequent parental psychological control. The limited extant research has highlighted the value of using longitudinal data to confirm whether Chinese parents will escalate or renounce the use of psychological control in the face of adolescents’ increasing affiliation with deviant peers. Based on the above-mentioned theories and literature review, longitudinal data were required to effectively examine whether deviant peer affiliation acts as a mediator and will explain the bidirectional association between parental psychological control and IGD.

### 1.3. The Current Study

The current study constructed an autoregressive cross-lagged model to investigate the longitudinal reciprocal relationships between parental psychological control, deviant peer affiliation, and IGD in a two-year longitudinal study among Chinese adolescents. The study employed a three-wave longitudinal design to allow a thorough understanding of the relationships between these variables. We have two hypotheses: (1) not only will parental use of psychological control elicit adolescent IGD, but also increased adolescent IGD will predict parental psychological control and (2) deviant peer affiliation will serve as a mediator in the path from parental psychological control to IGD, and the indirect effect of IGD on parental psychological control via deviant peer affiliation will also be significant.

## 2. Methods

### 2.1. Participants

This was a multisite longitudinal study; data for the present study were collected at one-year intervals: fall of seventh-grade, fall of eighth-grade, and fall of ninth-grade. Students voluntarily participated in the study during their classes. The baseline assessment included 1089 seventh-grade students (513 girls) aged between 9 and 14 years. After one year, the grade 7 students recruited in the first wave assessment were in grade 8 and took part in the second wave assessment. These students also participated in the third wave assessment in grade 9. The second wave of the study included 1024 eighth-grade students (94.03% of the original sample; 484 girls). The third wave included 908 ninth-grade students (83.38% of the original sample; 428 girls). Data for 908 adolescents (428 girls) who participated in all three assessments were ultimately included in the final analyses. A total of 181 students lacked data primarily because they transferred to a new school or dropped out of the study.

### 2.2. Procedure

Upon securing cooperation with the school authorities, using a convenience sampling approach, we invited students from four junior middle schools in the Guangdong province of southern China to participate in the longitudinal study. This survey was conducted in three large Chinese cities, namely Guangzhou, Dongguan, and Zhongshan, which have population sizes of 15.3059 million, 8.4645 million, and 3.38 million, respectively. Two junior middle schools in Guangzhou, one in Dongguan, and one in Zhongshan were selected. Cluster sampling was used to collect data; all seventh-grade students of the participating schools were invited to complete the baseline assessment survey. Prior to data collection, written informed consent was obtained from participants’ parents. The questionnaires were administered by trained data collectors in the participants’ classrooms during each assessment. Prior to each assessment, the data collectors informed participants that they could hand in the questionnaires at any time if they felt uncomfortable. Participants were also advised that the data collected would remain confidential. This study was approved by the Research Ethics Committee of Guangzhou University (NO. GZHU 2017012).

#### 2.2.1. Parental Psychological Control

Parental psychological control was assessed using the eight-item Chinese version [44] of the Parental Psychological Control questionnaire [19], which has been validated and has good reliability among Chinese adolescents [16,34,44]. The eight items reflected levels of parental psychological control (e.g., “My parents are always trying to change how I feel or think about things”) and responses were provided using a three-point scale ranging from 1 (*never*) to 3 (*frequently*). The mean score for the eight items was calculated, with higher scores indicating greater levels of parental psychological control. Cronbach’s αs ranged from 0.69 to 0.86 across the three waves of this study.

#### 2.2.2. Deviant Peer Affiliation

We used a 12-item questionnaire to assess deviant peer affiliation [23] that has demonstrated excellent reliability and validity with a sample of Chinese adolescents [45]. The participants were asked to report how many of their friends engaged in deviant behaviors during the past 6 months on a 5-point scale ranging from 1 (*never*) to 5 (*six or more times*) (e.g., “How many of your close friends were involved in fights in the past 6 months?”). The mean score for the 12 items was calculated, with higher scores indicating greater association with deviant friends. Cronbach’s αs ranged from 0.84 to 0.89 across the three waves of this study.

#### 2.2.3. IGD

IGD was measured using the 11-item Chinese version of the Internet Gaming Disorder questionnaire [46]. Participants were asked to report their degree of participation in Internet gaming during the past 6 months on a 3-point scale: 1 (*never*), 2 (*sometimes*), and 3 (*often*) (e.g., “Do you feel restless, frustrated, or irritable when you try to cut down or stop playing online games?”). Scores were recorded as: 0 = “never,” 0.5 = “sometimes,” and 1 = “frequently”. This scoring method allows participants who occasionally experienced symptoms to be taken into account, thus increasing accuracy [46]. The total score for the 11 items was calculated, with higher scores indicating higher levels of IGD. According to the criteria of Young and de Abreu [1], the cut-off score for identifying adolescents with IGD is 5 or more. Cronbach’s αs ranged from 0.76 to 0.85 across the three waves of this study.

#### 2.2.4. Covariates

The potential covariate effects of several variables, including sex, age, and family socioeconomic status [10,46], were controlled for. Gender was converted to a dummy variable (1 = male; 0 = female). Based on previously established methodology [47], socioeconomic status was calculated as the average of participants’ standardized scores for three items, including father’s and mother’s educational level (scale range: from 1 (unschooled) to 8 (doctor or above)) and family per capita monthly income (scale range: from 1 (≤¥1000) to 10 (≥¥9001)).

### 2.3. Statistical Analysis

We examined the differences between the participants who took part in all three measurements and those who dropped out of the study using chi-square tests and *t*-tests. In accordance with Cole and Maxwell’s [48] recommendations for assessing longitudinal mediation, we measured all variables at all three time points and specified autoregressive models. Path analysis was performed using Mplus 8.0. We estimated the longitudinal effects of parental psychological control and IGD using autoregressive cross-lagged models. Furthermore, we assessed the indirect effects of parental psychological control and IGD via deviant peer affiliation.

We used a stepwise method that allowed for nested model testing for each effect to determine which model exhibited the best fit to the data. Specifically, we began the analysis with a model that included only the autoregressive paths and concurrent correlations between study variables. In the second step, we included lagged paths with a 1-year interval in the model. In the third step, we included lagged paths with a 2-year interval in the model. In the fourth step, we analyzed and compared these parallel autoregressive models and constructed a reciprocal cross-lagged panel model with the best fit. Due to space limitations, the results section includes only the final model.

We performed a full information maximum-likelihood estimation procedure to account for missing data, using Mplus 8.0 [49]. In addition, all variables were standardized to reduce multicollinearity. As IGD scores were not normally distributed, bias-corrected bootstrap confidence intervals (CIs) with 1000 resamples were used to estimate the statistical significance of paths. Model fit was assessed using three standard indices: chi-square and normed chi-square (*χ*^2^/*df*), the comparative fit index (CFI), and the root mean squared error of approximation (RMSEA). According to Kline [50] and Hoyle [51], model fit is considered excellent with *χ*^2^/*df* of ≤3.00, CFI of ≥0.95, and RMSEA of ≤0.06.

## 3. Results

### 3.1. Descriptive Statistics and Correlations

According to the criteria of Young and Abreu [1], 5.95%, 8.37%, and 5.95% of the adolescents in the present sample were classified as IGD in grades 7, 8, and 9, respectively. This prevalence rate is similar to national Chinese data [11]. Chi-square tests and *t*-tests revealed no significant differences in any study variable between the participants who took part in all three measurements and those who dropped out of the study. At the time of baseline assessment, participants were aged between 9 and 14 years and the mean and standard deviation was 11.27 years (±1.54). Table 1 shows an overview of the means, standard deviations, and correlations between parental psychological control, deviant peer affiliation, and IGD across the three waves of the study. Bivariate correlation analysis indicated that most correlations were statistically significant and occurred in the expected direction.

### 3.2. Cross-Lagged Association between Parental Psychological Control and IGD

**Figure 1:**
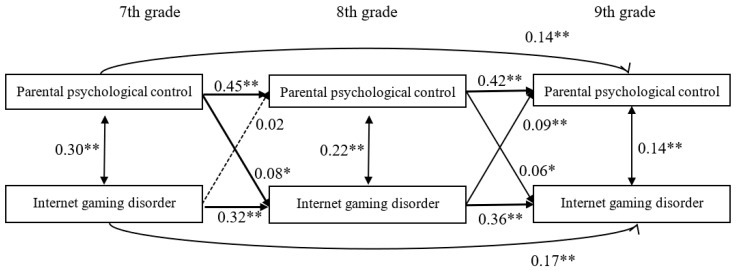
Model of the longitudinal association between parental psychological control and Internet gaming disorder. *Note.* The dotted line indicates a non-significant relationship. The path coefficients are standardized coefficients. * *p* < 0.05, ** *p* < 0.01.

Model 1 (Figure 1) examined the cross-lagged effect between parental psychological control and IGD and fit the data well; *χ*^2^(2) = 0.14, *χ*^2^/*df* = 0.07, CFI = 1.00, and RMSEA = 0.00. The path shown in Figure 1 indicated that parental psychological control observed in the seventh grade significantly predicted IGD in the eighth grade (*β* = 0.08, *p* < 0.05), and parental psychological control observed in the eighth grade significantly predicted IGD in the ninth grade (*β* = 0.06, *p* < 0.05). Moreover, IGD observed in the eighth grade significantly predicted parental psychological control in the ninth grade (*β* = 0.09, *p* < 0.01). However, the predictive effect of IGD observed in the seventh grade on parental psychological control in the eighth grade was non-significant (*β* = 0.02, *p* = 0.55).

Moreover, of the paths between gender, age, socioeconomic status (SES), and each of the variables in the model, the following were significant. First, gender (*β* = 0.16, *p* < 0.01) and age (*β* = 0.10, *p* < 0.05) significantly predicted IGD in the eighth grade. Second, gender (*β* = 0.19, *p* < 0.01) and SES (*β* = −0.09, *p* < 0.01) significantly predicted IGD in the 9th grade.

### 3.3. Longitudinal Indirect Effect of Deviant Peer Affiliation on the Association between Parental Psychological Control and IGD

**Figure 2:**
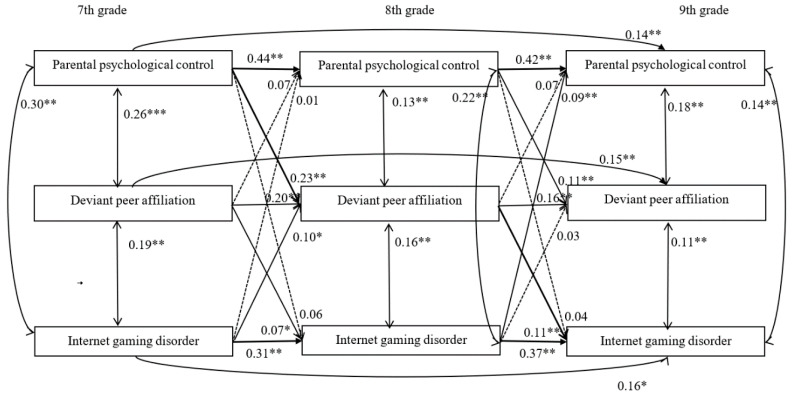
Model of the indirect role of deviant peer affiliation in the association between parental psychological control and Internet gaming disorder. Note: dotted lines indicate non-significant relationships. The path coefficients are standardized coefficients. * *p* < 0.05, ** *p* < 0.01, *** *p* < 0.001.

Model 2 (Figure 2) examined the longitudinal indirect role of deviant peer affiliation in the association between parental psychological control and IGD. Model 2 exhibited an excellent fit to the data, *χ*^2^(6) = 10.53, *χ*^2^/*df* = 1.76, CFI = 1.00, RMSEA = 0.03. As depicted by the central downward paths in Model 2, parental psychological control observed in the 7th grade predicted deviant peer affiliation in the 8th grade (*β* = 0.23, *p* < 0.01), which predicted IGD in the 9th grade (*β* = 0.11, *p* < 0.01). Bias-corrected bootstrapping revealed a significant indirect effect of parental psychological control on IGD via deviant peer affiliation: indirect effect = 0.13, 95% CI = 0.03–0.24. Moreover, although IGD observed in the 7th grade predicted deviant peer affiliation in the 8th grade (*β* = 0.10, *p* < 0.05), deviant peer affiliation in the 8th grade did not significantly predict parental psychological control in the 9th grade (*β* = 0.07, *p* = 0.06). Bias-corrected bootstrapping revealed a non-significant indirect effect of IGD on parental psychological control via deviant peer affiliation: indirect effect = 0.002, 95% CI = 0.00–0.01.

Moreover, of paths between gender, age, socioeconomic status (SES), and each of the variables in the model, the following were significant. First, gender significantly predicted deviant peer affiliation in the 8th grade (*β* = 0.07, *p* < 0.05), and gender (*β* = 0.16, *p* < 0.01) and age (*β* = 0.09, *p* < 0.05) significantly predicted IGD in the 8th grade. Second, gender (*β* = 0.09, *p* < 0.01), age (*β* = 0.10, *p* < 0.05), and SES (*β* = 0.09, *p* < 0.05) significantly predicted deviant peer affiliation in the ninth grade, and gender (*β* = 0.19, *p* < 0.01) and SES (*β* = −0.09, *p* < 0.01) significantly predicted IGD in the ninth grade.

## 4. Discussion

The current study was conducted in a Chinese context and its objective was to explore the causal and bidirectional relationships among parental psychological control, deviant peer affiliation, and IGD using a three-wave data model. The results indicate bidirectional effects of parental psychological control and IGD. Furthermore, deviant peer affiliation exerted a mediating effect on the path from parental psychological control to IGD. However, the indirect effect of IGD on parental psychological control via deviant peer affiliation was non-significant. These findings suggest that parental and peer-related factors could increase the risk of IGD in adolescence.

### 4.1. Longitudinal Direct Association

Our results indicate that there was a reciprocal direct effect; specifically, the effect of parental psychological control’s prediction of adolescent IGD was stable, and the effect of IGD on parental psychological control was also significant but not stable over time. This finding is consistent with the self-determination theory [20] and the concept of psychological control, which undermines adolescents’ development of autonomy and competence, possibly leading adolescents to indulge in online games as a means of escaping from a stressful reality [52]. The current findings not only corroborate those of previous empirical research examining parenting, which indicated that psychological control exerted a negative influence on child development [11,13], but also revealed that psychological control is a strong predictor of IGD, consistent with previous research conducted in Hong Kong indicating that psychological control exerts a long-lasting effect on adolescent Internet addiction [14].

In addition, we observed that IGD predicted increases in subsequent parental psychological control. These findings suggest that when adolescents exhibit inappropriate behavior (e.g., IGD), their parents may take action (e.g., increases in psychological control) to reduce the occurrence of inappropriate behavior, which is consistent with the social coercion theory [26,27]. At the same time, the effect of IGD on subsequent parental psychological control was not stable over time; only IGD observed in the eighth grade positively predicted psychological control in the ninth grade. One possible reason is that Chinese parents are most concerned about their children’s academic performance and expect their children to concentrate all their energy and time on their studies, especially in the ninth grade, faced with advancement from junior to senior high school [30,31]. However, adolescents who are fascinated with online games spend most of their time playing games [1], which, in turn, increases parental use of psychological control to force a reduction in time spent playing online games.

### 4.2. Longitudinal Indirect Association

The current findings demonstrate that deviant peer affiliation is an important underlying mediation mechanism that explains how parental psychological control exerts an effect on IGD among adolescents. Specifically, adolescents who experience parental psychological control are likely to affiliate with deviant peers, which predicts high levels of IGD over time. This result suggests that a mismatch of context between adolescents’ basic psychological needs and parental psychological control could increase adolescents’ willingness to remain with their peers, ultimately leading to the development of aggressive behavior. We found the effect of parental psychological control on deviant peer affiliation to be stable in the current study, in line with earlier findings that parental psychological control is a critical risk factor for deviant peer affiliation [34,35]. Adolescents who are constantly subjected to parental psychological control are deprived of the right to express emotions and exert independence. Consequently, they are inclined to report low self-esteem and impaired emotion regulation, leading to problems in interpersonal relationships [24], which increases the likelihood of affiliation with deviant peers. In addition, adolescents whose basic psychological needs are not being met are more likely to increase their affiliation with deviant peers in search of peer support and psychological security [23].

It has been highlighted that deviant peer affiliation is a crucial factor in the etiology of externalizing problems (e.g., IGD) [23,45], which is consistent with the current finding that adolescents’ affiliation with deviant peers in the seventh grade predicted IGD in the eighth grade, while deviant peer affiliation in the eighth grade predicted IGD in the ninth grade. These results suggest the predictive role of deviant peer affiliation on subsequent IGD to be stable over time. Under the influence of deviant peers, adolescents could adapt their attitudes toward IGD or attempt to play online games to integrate into peer groups in deviant peer clusters [53]. In addition, peer pressure can increase the likelihood that adolescents will develop IGD, particularly with frequent communication with peers as a means of integrating into peer groups. According to the longitudinal tracking data, this finding suggests that inappropriate parenting (e.g., parental psychological control) affects adolescents’ propensity to affiliate with deviant peers, amplifying the risk of problematic behavior (e.g., IGD).

Furthermore, we also assessed the mediating role of deviant peer affiliation in the path from IGD to parental psychological control; however, IGD did not exert an effect on parental psychological control via deviant peer affiliation. Specifically, deviant peer affiliation in the eighth grade did not predict parental psychological control in the ninth grade, and the path from deviant peer affiliation in the seventh grade to parental psychological control in the eighth grade was also non-significant. This finding is contrary to the coercion theory, which holds that parents tend to respond to unwanted behaviors (e.g., affiliation with deviant peers) by increasing their psychological control [27]. One possible explanation is that parents’ perceived lack of success in achieving psychological control ultimately leads them to give up their psychological control attempts. As our current findings demonstrate, psychological control robustly predicts future increases in deviant peer affiliation. Moreover, Chinese culture emphasizes a collectivist orientation [54]; therefore, parents may be more concerned about the potential negative influence of their child’s affiliation with deviant peers. Thus, parents may realize that psychological control might not be an effective method of reducing adolescent problem behaviors and gradually change their parenting style [55]. Another possible explanation is that adolescents affiliated with deviant peers develop a false understanding of norm-violating behavior and deem the acts that deviate from or violate accepted social norms to be normal due to deviancy training [56]. In turn, these attitudes may lead adolescents affiliated with deviant peers to rebel against their parents as they attempt to gradually rid themselves of parental psychological control.

The current results demonstrate that IGD predicts subsequent deviant peer affiliation; IGD in adolescents has previously been significantly associated with antisocial behavior, emotional dysregulation, and low self-esteem [10], which could result in rejection by mainstream peers, and adolescents who frequently experience peer rejection are likely to affiliate with deviant peers via default selection [57]. A one-year longitudinal study revealed that adolescents who were gradually forced out of conventional peer groups affiliated with deviant peers via default selection because of a lack of viable alternatives [41]. In addition, adolescents with IGD select deviant peer groups via homophily selection processes because they perceive similar behavioral tendencies [40]. Unexpectedly, the longitudinal paths from IGD to deviant peer affiliation were unstable. Specifically, IGD observed in the seventh grade significantly positively predicted deviant peer affiliation in the eighth grade; however, IGD in the eighth grade did not predict deviant peer affiliation in the ninth grade. One possible explanation for this finding is that students in the seventh grade had just entered junior high school and left a familiar environment, which stimulated their desire for friendship. During this period, adolescents with IGD could easily select deviant peer groups. However, as they entered the eighth grade, they had made friends, and a decreased need for friends could reduce the effect of IGD on deviant peer affiliation over time.

### 4.3. Limitations and Future Directions

There are several limitations to the current study. First, all data were collected via self-report questionnaires, and the findings were likely influenced by common method variance and the social desirability bias. Nonetheless, some research suggests that self-reports of parental psychological control can provide valuable information [22], as adolescents have a better sense of the true extent of their parents’ psychological control. Future research should incorporate data from multiple informants (e.g., parents and peers) and different methods (e.g., interviews). Second, in longitudinal psychological research, repeated assessment could lead to repeated and biased responses by respondents. However, in the current study, participants answered questions truthfully based on their current situations (e.g., parenting characteristics and IGD) and the data were tracked for two years; therefore, memory effects are unlikely to have affected the results. Third, caution should be taken when extending the results of this study to other parenting dimensions and styles, and further longitudinal research is required to determine whether deviant peer affiliation mediates the effects of different parenting styles on IGD. Furthermore, the cross-lagged models were based on the aggregated data from each wave. Future studies should consider exploring the moderating effect of individual characteristics on direct or mediating pathways from parental psychological control to IGD across time. Fourth, the study participants in Guangdong province were selected through convenience sampling; this type of sampling procedure might introduce sampling bias, which might affect the validity of the study, so we should be cautious in generalizing our results to other populations. Future studies should use other samples from multiple regions to verify the association.

## 5. Conclusions

The current results extend those of previous studies and indicate that parental psychological control is a clear risk factor for adolescent IGD, and the reciprocal effect of parental psychological control and IGD was significant. Specifically, parental psychological control robustly predicted subsequent IGD, and high levels of IGD in the eighth grade predicted increased parental psychological control in the ninth grade. Moreover, the indirect longitudinal path from parental psychological control to IGD via deviant peer affiliation was significant; however, the inverse association was non-significant. These findings enhance the understanding of the reciprocal effect of parental psychological control and adolescent IGD in the Chinese context.

## Figures and Tables

**Table 1:** Descriptive statistics and correlations for all variables (*N* = 908).

Variable	1	2	3	4	5	6	7	8	9
1. PPC at 7th grade	1.00								
2. PPC at 8th grade	0.45 **	1.00							
3. PPC at 9th grade	0.34 **	0.50 **	1.00						
4. DPA at 7th grade	0.24 **	0.15 **	0.09 *	1.00					
5. DPA at 8th grade	0.31 **	0.25 **	0.19 **	0.30 **	1.00				
6. DPA at 9th grade	0.15 **	0.18 **	0.25 **	0.24 **	0.25 **	1.00			
7. IGD at 7th grade	0.30 **	0.15 **	0.14 **	0.15 **	0.21 **	0.17 **	1.00		
8. IGD at 8th grade	0.19 **	0.26 **	0.23 **	0.13 **	0.26 **	0.14 **	0.40 **	1.00	
9. IGD at 9th grade	0.17 **	0.18 **	0.24 **	0.15 **	0.24 **	0.25 **	0.37 **	0.49 **	1.00
*Mean*	1.43	1.47	1.46	1.27	1.29	1.17	1.47	1.38	1.00
*SD*	0.33	0.41	0.43	0.52	0.53	0.41	1.62	1.78	1.57

Note: PPC = parental psychological control; DPA = deviant peer affiliation; IGD = Internet gaming disorder. * *p* < 0.05; ** *p* < 0.01.

## References

[1] Young K.S., de Abreu C.N. (2011). Internet Addiction: A Handbook and Guide to Evaluation and Treatment.

[2] Sussman C.J., Harper J.M., Stahl J.L., Weigle P. (2018). Internet and video game addictions: Diagnosis, epidemiology, and neurobiology. Child Adolesc. Psychiatr. Clin. N. Am..

[3] Feng W., Ramo D.E., Chan S.R., Bourgeois J.A. (2017). Internet gaming disorder: Trends in prevalence 1998–2016. Addict. Behav..

[4] Long J., Liu T., Liu Y., Hao W., Maurage P., Billieux J. (2018). Prevalence and correlates of problematic online gaming: A systematic review of the evidence published in Chinese. Curr. Addict. Rep..

[5] Association A.P. (2013). Diagnostic and Statistical Manual of Mental Disorders DSM-5^®^.

[6] Casey B.J., Jones R.M., Hare T.A. (2008). The adolescent brain. Ann. N. Y. Acad. Sci..

[7] Brand M., Young K.S., Elaier C. (2014). Prefrontal control and internet addiction: A theoretical model and review of neuropsychological and neuroimaging findings. Front. Hum. Neurosci..

[8] Stavropoulos V., Kuss D., Griffiths M.D., Wilson P., Motti-Stefanidi F. (2017). MMORPG gaming and hostility predict Internet Addiction symptoms in adolescents: An empirical multilevel longitudinal study. Addict. Behav..

[9] Griffiths M.D., Király O., Pontes H.M., Demetrovics Z., Starcevic V., Aboujaoude E. (2015). An overview of problematic gaming. Mental Health in the Digital Age: Grave Dangers, Great Promise.

[10] Mihara S., Higuchi S. (2017). Cross-sectional and longitudinal epidemiological studies of Internet gaming disorder: A systematic review of the literature. Psychiatry Clin. Neurosci..

[11] Yang X., Jiang X., Mo P.K.H., Cai Y., Ma L., Lau J.T.F. (2020). Prevalence and interpersonal correlates of internet gaming disorders among Chinese Adolescents. Int. J. Environ. Res. Public Health.

[12] Wong H.Y., Mo H.Y., Potenza M.N., Chan M.N.M., Lau W.M., Chui T.K., Pakpour A.H., Lin C.-Y. (2020). Relationships between severity of internet gaming disorder, severity of problematic social media use, sleep quality and psychological distress. Int. J. Environ. Res. Public Health.

[13] Li X., Li D., Newman J. (2013). Parental behavioral and psychological control and problematic internet use among Chinese adolescents: The Mediating role of self-control. Cyberpsychol. Behav. Soc. Netw..

[14] Shek D.T.L., Zhu X., Ma C.M.S. (2018). The influence of parental control and parent-child relational qualities on adolescent internet addiction: A 3-year longitudinal study in Hong Kong. Front. Psychol..

[15] Bell R.Q. (1968). A reinterpretation of the direction of effects in studies of socialization. Psychol. Rev..

[16] Chen Y., Zhu J., Yu C., Wang M., Zhu Y., Zhang W. (2019). The explanatory mechanism of child impulsivity in the bidirectional associations between parental psychological control and child physical aggression. J. Child Fam. Stud..

[17] Ho D.Y. (1994). Filial piety, authoritarian moralism, and cognitive conservatism in Chinese societies. Genet. Soc. Gen. Psychol. Monogr..

[18] Chao R.K. (1994). Beyond parental control and authoritarian parenting style: Understanding Chinese parenting through the cultural notion of training. Child Dev..

[19] Barber B.K. (1996). Parental psychological control: Revisiting a neglected construct. Child Dev..

[20] Ryan R.M., Deci E.L. (2000). Self-determination theory and the facilitation of intrinsic motivation, social development, and well-being. Am. Psychol..

[21] Przybylski A.K., Rigby C.S., Ryan R.M. (2010). A motivational model of video game engagement. Rev. Gen. Psychol..

[22] Barber B.K., Harmon E.L., Barber B. (2002). Violating the self: Parental psychological control of children and adolescents. Intrusive Parenting: How Psychological Control Affects Children and Adolescents.

[23] Zhu J., Zhang W., Yu C., Bao Z. (2015). Early adolescent Internet game addiction in context: How parents, school, and peers impact youth. Comput. Hum. Behav..

[24] Song J.J., Li D.P., Gu C.H., Zhao L.Y., Bao Z.Z., Wang Y.H. (2014). Parental control and adolescents’ problematic Internet use: The mediating effect of deviant peer affiliation. Psychol. Dev. Educ..

[25] Koo H.J., Kwon J.-H. (2014). Risk and protective factors of internet addiction: A meta-analysis of empirical studies in Korea. Yonsei Med. J..

[26] Patterson G.R. (1982). Coercive Family Processes.

[27] Reid J.B., Patterson G.R., Snyder J. (2002). Antisocial Behavior in Children and Adolescents: A Developmental Analysis and Model for Intervention.

[28] Su B., Yu C., Zhang W., Su Q., Zhu J., Jiang Y. (2018). Father–child longitudinal relationship: Parental monitoring and internet gaming disorder in Chinese Adolescents. Front. Psychol..

[29] Koning I.M., Peeters M., Finkenauer C., Eijnden R.J.J.M.V.D. (2018). Bidirectional effects of Internet-specific parenting practices and compulsive social media and Internet game use. J. Behav. Addict..

[30] Chen C., Uttal D.H. (1988). Cultural values, parents’ beliefs, and children’s achievement in the United States and China. Hum. Dev..

[31] Zhu J., Yu C., Bao Z., Jiang Y., Zhang W., Chen Y., Qiu B., Zhang J. (2017). Deviant peer affiliation as an explanatory mechanism in the association between corporal punishment and physical aggression: A longitudinal study among Chinese Adolescents. J. Abnorm. Child Psychol..

[32] Pires P., Jenkins J.M. (2006). A growth curve analysis of the joint influences of parenting affect, child characteristics and deviant peers on adolescent illicit drug use. J. Youth Adolesc..

[33] Soenens B., Vansteenkiste M., Smits I., Lowet K., Goossens L. (2007). The role of intrusive parenting in the relationship between peer management strategies and peer affiliation. J. Appl. Dev. Psychol..

[34] Tian Y., Yu C., Lin S., Lu J., Liu Y., Zhang W. (2019). Parental psychological control and adolescent aggressive behavior: Deviant peer affiliation as a mediator and school connectedness as a moderator. Front. Psychol..

[35] Keijsers L., Branje S., Hawk S.T., Schwartz S.J., Frijns T., Koot H.M., Van Lier P., Meeus W. (2011). Forbidden friends as forbidden fruit: Parental supervision of friendships, contact with deviant peers, and adolescent delinquency. Child Dev..

[36] Beard K.W., Young K.S., de Abreu C.N. (2011). Working with Adolescents Addicted to the Internet. Internet Addiction: A Handbook and Guide to Evaluation and Treatment.

[37] Ko C.-H., Yen J.-Y., Yen C., Chen C.-S., Weng C., Chen C.C. (2008). The association between internet addiction and problematic alcohol use in adolescents: The problem behavior model. CyberPsychol. Behav..

[38] Cutrín O., Maneiro L., Sobral J., Gómez-Fraguela J. (2018). Longitudinal effects of parenting mediated by deviant peers on violent and non-violent antisocial behaviour and substance use in adolescence. Eur. J. Psychol. Appl. Leg. Context.

[39] Veenstra R., Dijkstra J.K., Laursen B., Collins W.A. (2011). Transformations in adolescent peer networks. Relationship Pathways: From Adolescence to Young Adulthood.

[40] Dishion T.J., Ha T., Véronneau M.-H. (2012). An ecological analysis of the effects of deviant peer clustering on sexual promiscuity, problem behavior, and childbearing from early adolescence to adulthood: An enhancement of the life history framework. Dev. Psychol..

[41] Jiang Y., Yu C., Zhang W., Bao Z., Zhu J. (2016). Peer victimization and substance use in early adolescence: Influences of deviant peer affiliation and parental knowledge. J. Child Fam. Stud..

[42] Sijtsema J.J., Lindenberg S., Veenstra R. (2010). Do they get what they want or are they stuck with what they can get? Testing homophily against default selection for friendships of highly aggressive boys. The trails study. J. Abnorm. Child Psychol..

[43] Werner N.E., Crick N.R. (2004). Maladaptive peer relationships and the development of relational and physical aggression during middle childhood. Soc. Dev..

[44] Yu C., Wu Q., Zhang W. (2017). The association between parental psychological control and adolescent depression: The moderation of parenthood. J. Lingnan Norm. Univ..

[45] Lin S., Yu C., Chen J., Zhang W., Cao L., Liu L. (2020). Predicting adolescent aggressive behavior from community violence exposure, deviant peer affiliation and school engagement: A one-year longitudinal study. Child. Youth Serv. Rev..

[46] Yu C., Li X., Zhang W. (2015). Predicting adolescent problematic online game use from teacher autonomy support, basic psychological needs satisfaction, and school engagement: A 2-year longitudinal study. Cyberpsychol. Behav. Soc. Netw..

[47] Veenstra R., Lindenberg S., Oldehinkel A.J., De Winter A.F., Ormel J. (2006). Temperament, environment, and antisocial behavior in a population sample of preadolescent boys and girls. Int. J. Behav. Dev..

[48] Cole D.A., Maxwell S.E. (2003). Testing mediational models with longitudinal data: Questions and tips in the use of structural equation modeling. J. Abnorm. Psychol..

[49] Enders C.K., Bandalos D.L. (2001). The relative performance of full information maximum likelihood estimation for missing data in structural equation models. Struct. Equ. Model. A Multidiscip J..

[50] Kline R.B. (2011). Principals and Practices of Structural Equation Modeling.

[51] Hoyle R.H. (2012). Handbook of Structural Equation Modeling.

[52] Wu C.S.T., Wong H.T., Yu K.F., Fok K.W., Yeung S.M., Lam C.H., Liu K.M. (2016). Parenting approaches, family functionality, and internet addiction among Hong Kong adolescents. BMC Pediatr..

[53] Akers R.L., Krohn M.D., Lanza-Kaduce L., Radosevich M. (1979). Social learning and deviant behavior: A specific test of a general theory. Am. Sociol. Rev..

[54] Chen X., Comunian A.L., Gielen U.P. (2000). Growing up in a collectivistic culture: Socialization and socio-emotional development in Chinese children. International Perspectives on Human Development Lengerich: Pabst Science.

[55] Pinquart M. (2017). Associations of parenting dimensions and styles with externalizing problems of children and adolescents: An updated meta-analysis. Dev. Psychol..

[56] Dishion T.J., Spracklen K.M., Andrews D.W., Patterson G.R. (1996). Deviancy training in male adolescent friendships. Behav. Ther..

[57] Rudolph K.D., Lansford J.E., Agoston A.M., Sugimura N., Schwartz D., Dodge K.A., Pettit G.S., Bates J.E. (2013). Peer victimization and social alienation: Predicting deviant peer affiliation in middle school. Child Dev..

